# Quantitative Analysis of SPECT-CT Data in Metastatic Breast Cancer Patients—The Clinical Significance

**DOI:** 10.3390/cancers14020273

**Published:** 2022-01-06

**Authors:** Mirela Gherghe, Mario-Demian Mutuleanu, Adina Elena Stanciu, Ionela Irimescu, Alexandra Lazar, Xenia Bacinschi, Rodica Maricela Anghel

**Affiliations:** 1 Nuclear Medicine Department, University of Medicine and Pharmacy Carol Davila Bucharest, 050474 Bucharest, Romania; mirela.gherghe@umfcd.ro; 2 Nuclear Medicine Department, Institute of Oncology “Prof.Dr. Alexandru Trestioreanu”, 022328 Bucharest, Romania; ionelanicoleta.irimescu@gmail.com (I.I.); alexandra-maria.lazar@rez.umfcd.ro (A.L.); 3 Carcinogenesis and Molecular Biology Department, Institute of Oncology “Prof.Dr. Alexandru Trestioreanu”, 022328 Bucharest, Romania; adinaelenastanciu@yahoo.com; 4 Oncology Department, University of Medicine and Pharmacy Carol Davila Bucharest, 050474 Bucharest, Romania; medicina.nucleara@iob.ro (X.B.); rodicamanghel@gmail.com (R.M.A.); 5 Radiotherapy Department, Institute of Oncology “Prof.Dr. Alexandru Trestioreanu”, 022328 Bucharest, Romania

**Keywords:** quantitative analysis, SPECT-CT, SUVmax, bone metastatic lesions, bone degenerative lesions

## Abstract

**Simple Summary:**

Breast cancer represents one of the most common cancers diagnosed in female patients, with up to 75% of the patients with stage IV breast cancer developing metastatic bone lesions. Early detection of bone metastasis and differentiating them from degenerative lesions using molecular imaging techniques, such as SPECT-CT, is important for therapeutic purposes and patient follow-up. This study was conducted to determine if the quantitative analysis of the data acquired by performing SPECT-CT scans can help in differentiating between metastatic lesions and degenerative bone disease. In 70 female patients, we identified the radiotracer uptake in metastatic and degenerative bone lesions and determined the diagnostic accuracy of the SPECT-CT quantitative analysis in differentiating between the two types of lesions. The results provided valuable information that can improve the diagnostic accuracy of metastatic bone lesions and treatment response evaluation in breast cancer patients.

**Abstract:**

Purpose: To assess the potential added value of the SPECT-CT quantitative analysis in metastatic breast cancer lesions detection and differentiation from degenerative lesions. Methods: This prospective monocentric study was conducted on 70 female patients who underwent SPECT-CT bone scans using ^99m^Tc–HDP that identified the presence of metastatic bone lesions and degenerative lesions in each patient. Once the lesions were identified, a quantitative analysis of radiotracer uptake was conducted. The highest one to five SUVmax values for both metastatic and degenerative bone lesions were identified in each patient and the data were then statistically analyzed. Results: The SUVmax value was significantly higher in metastatic bone lesions than in degenerative lesions (*p* < 0.001). The diagnostic accuracy of SPECT-CT quantitative data analysis revealed a sensitivity of 91.5% and a specificity of 93.3% at a cut-off value of the SUVmax of 16.6 g/mL. Conclusion: Quantitative analysis performed using SPECT-CT data can improve the diagnostic accuracy in differentiating between metastatic bone lesions and degenerative lesions, thus leading to appropriate treatment and better follow-up in metastatic breast cancer patients.

## 1. Introduction

Breast cancer represents one of the most common cancers diagnosed in female patients, with an estimated incidence of 11.7%, according to GLOBOCAN data from 2020, representing as many as 2.2 million new cases worldwide [1]. Previous studies have shown that the prevalence of bone metastases varies between 50% and 75% in patients with metastatic breast cancer [2,3,4,5,6,7]. The use of molecular imaging techniques, such as single photon emission computed tomography (SPECT) and positron emission tomography (PET), has led to improved diagnostic accuracy. Both imaging modalities rely on a tracer principle in which a predetermined quantity of a radiotracer is administered to the patient in order to assess the metabolic activity in certain regions of the body. In practice, the acquired images are either visually assessed or can be quantitatively evaluated using complex algorithms, thus providing a more standardized interpretation by expressing the radiotracer activity in absolute units [8]. To perform an accurate quantitative analysis of regions of interest (ROI), some obstacles, such as photon attenuation, photon scatter, and partial volume effect, needed to be overcome. Unlike for PET imaging, where solutions were identified several decades ago, the SPECT technological process has been proven to be a slow and complex task in this direction [8]. SPECT has been conventionally regarded in a nonquantitative manner, the acquired data being assessed using relative intensity values and not absolute units [8,9,10]. With the emergence of hybrid imaging techniques (SPECT-CT and PET-CT), transmission scanning has been proven to be useful for achieving an accurate attenuation correction map [11,12]. Because the incorporation of the measured attenuation map in the image reconstruction algorithm cannot be done in a straightforward manner, further development of iterative reconstruction methods had to be conducted, culminating with the development of OSEM (ordered subset expectation maximization) algorithm; moreover, the discovery of the new scatter correction algorithms opened up the possibility to perform accurate measurements of the radiotracer activity in SPECT-CT studies [13]. Given the potential to acquire dual or multiple radionuclide studies, its wide range of applications, combined with the quantitative accuracy that can be obtained using CT information, there has been a growing interest in SPECT-CT, which now offers a viable, low-cost alternative to PET-CT [14].

The worldwide acceptance of SPECT-CT scanners and the development in imaging reconstruction techniques have made the clinical use of the SPECT-CT quantitative analysis a viable option [15,16]. It is well known that every imaging technique has its strengths and weaknesses that mainly depend on the underlying principle used for image acquisition. Hybrid imaging can be used to combine the strengths of the individual component while compensating for their weaknesses. SPECT-CT, PET-CT, or PET-MRI represent well-established examples in clinical practice [17]. The study performed by Arvola et al. determined the correlation between the SUV values measured for ^99m^Tc –HDP SPECT-CT and ^18^F-NaF PET-CT, and the data showed strong correlations between the two methods, thus indicating the feasibility of using SPECT-CT SUVs in clinical practice [18]. The feasibility of SPECT-CT in performing quantitative analysis of bone lesions has also been demonstrated in other studies that reported SUVs values of the radiotracer uptake in bone lesions [18,19,20,21,22]. Moreover, the sensitivity of SPECT-CT in detecting bone metastatic lesions, as reported in previous studies compared to whole body scintigraphy, ranges between 89–95% compared to 59–93% with a specificity of 82-95% compared to 59–74%, respectively [23,24,25]. This study was conducted in order to determine whether quantitative analysis of the data acquired by SPECT-CT imaging can help to differentiate between metastatic lesions and degenerative bone disease.

## 2. Materials and Methods

### 2.1. Patients

**Figure 1:**
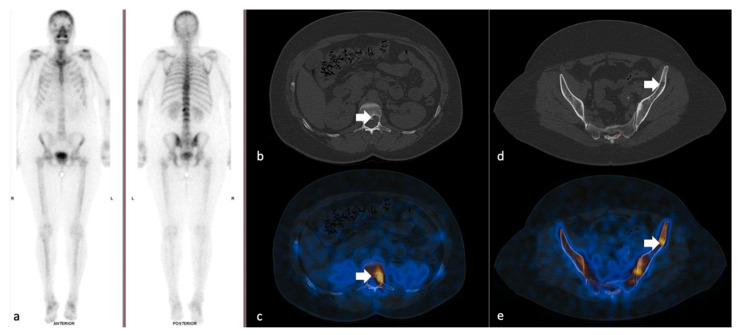
47 year-old female patient with breast carcinoma who underwent quantitative analysis SPECT-CT scanning: (**a**) whole body scan showing faint uptake in the L2 vertebral body; (**b**,**c**) high uptake in the L2 osteolytic lesion; and (**d**,**e**) osteolytic lesion on the iliac bone undefined on the planar scintigraphy showing increased radiotracer uptake.

This is a prospective study in which every patient had to fully understand and agree to the informed consent, signed prior to study entry. The study was approved by the local ethics committee and was conducted from the 1st of October 2020 to the 1st of June 2021 at the Department of Nuclear Medicine of the National Institute of Oncology “Prof. Dr. Alexandru Trestioreanu” in Bucharest, Romania. The reference population in our study was represented by female patients diagnosed with breast cancer and referred for bone scan examination. The study population included 70 female patients with a mean age of 59.36 ± 11.45 years who underwent whole body scintigraphy and quantitative assessment using dedicated software technology for SPECT-CT acquisition data. For each patient, after the initial whole-body scan, two experienced physicians analyzed the images and decided if the SPECT-CT scanning was required. The main criteria for SPECT-CT scanning referral was represented by the presence of lesions with high uptake on the whole-body bone scan, as it was difficult to discriminate between metastatic and degenerative lesions (Figure 1).

The patients were included in the study based on the following criteria:

○ breast carcinoma confirmed through biopsy in different clinical scenarios: initial staging in symptomatic patients or initial staging for patients with clinical stage III and IV, restaging for patients with new bone pain or increase in alkaline phosphatase level, and restaging for nonosseous recurrence [26]
○ availability of the data regarding the time of injection, the injected activity, residual activity measurement, and time of the acquisition
○ access to the patient’s medical history
○ at least one metastatic and degenerative lesion in the SPECT-CT field of view (FOV)

### 2.2. Image Acquisition and Reconstruction

**Figure 2:**
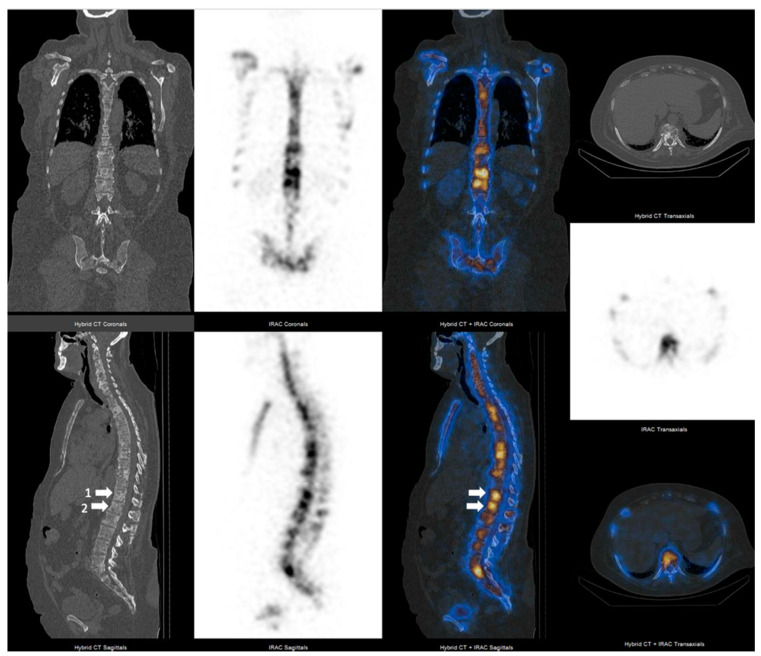
62 year-old breast cancer female patient with grade 2 ductal invasive carcinoma presenting with pain and elevated alkaline phosphatase, with high radiotracer uptake in multiple lytic bone metastatic lesions (1–2 = lytic lesions with the highest SUVmax value localized in T12-L1 vertebrae).

**Figure 3:**
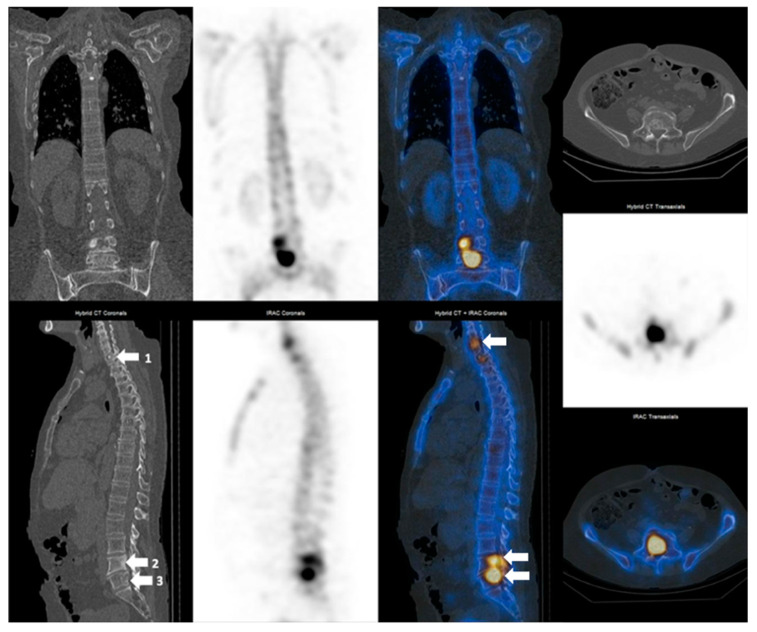
69 year-old breast cancer female patient with grade 2 invasive ductal carcinoma. First SPECT-CT scanning revealing both osteolytic and osteoblastic metabolically active metastatic lesions (1—degenerative lesion SUVmax 24.1 g/mL T1 vertebra, 2—osteoblastic lesion SUVmax 27.7 g/mL L4 vertebra, 3—osteolytic lesion SUVmax 76.9 g/mL L5 vertebra).

The patients received an intravenous injection of 673.61 ± 56.64 MBq of Tc^99m^-HDP. The SPECT-CT scans were performed 176.32 ± 34.47 min after the injection of the radiotracer using a SPECT-CT scanner (GE Discovery D670) with a sensitivity of 160 counts per minute, using low-energy, high-resolution collimators. We implemented the SPECT-CT protocol according to the European guidelines: 128 by 128 matrix size, step and shoot mode, 60 steps, with 20 s per step. For scatter correction, dual-energy window with 140 KeV peak energy ± 10 KeV and 120 ± 5 KeV was chosen. After completion of the SPECT acquisition, maintaining the patient in the same position, a low-dose CT scan was performed using 120 kV and 30 mA using dose modulation (smart scan; General Electric). CT data were acquired with 3.75 mm slice thickness and then reconstructed in 1.25 mm slice thickness data using bone enhancement filters provided by the vendor software (BONE PLUS reconstruction filter) to reduce the number of unidentified lesions (Figure 2 and Figure 3). The reconstruction of the SPECT-acquired data was performed using the ordered subset expectation maximization (OSEM) iterative reconstruction algorithm with eight subsets and 10 iterations, resolution recovery, scatter correction, and attenuation correction based on the attenuation correction linear coefficient map which resulted from the transformation of the CT data in the SPECT reconstruction algorithm compatible data.

### 2.3. Image Interpretation and Quantitative Assessment

**Figure 4:**
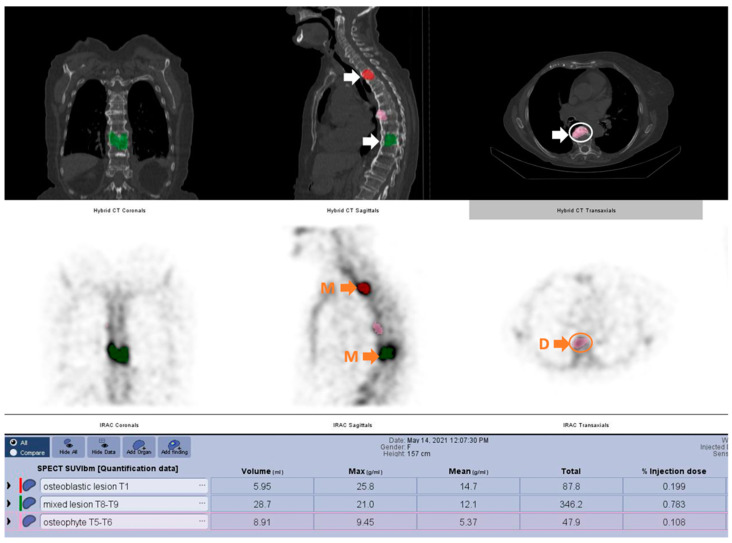
49 year-old female patient with invasive ductal carcinoma grade 3, presenting back pain in the thoracic region due to metabolically active osteoblastic, mixed metastatic lesions (T1 osteoblastic and T8-T9 mixed) and degenerative lesions (T5–T6) (D = degenerative, M = metastatic).

The acquired images were interpreted independently by two experienced physicians on a dedicated workstation for imaging diagnosis (GE Xeleris 4.0). Discordant results reached a consensus within a joint meeting. After the qualitative assessment of images, the areas with increased radiotracer activity were categorized as either metastases or degenerative lesions. Several lesions were evaluated, with a maximum of five lesions, each one with a different SUVmax value. The delineation of the volume of interest (VOI) was performed using the quantitative analysis software tool provided by the camera’s vendor (GE Q. Volumetrix) (Figure 4). The contours of the VOIs were drawn semi-automatic using one of the methods made available by the vendor software. The SUVmax based on lean body mass was the method of choice in calculating the radiotracer uptake. After the identification and quantitative analysis of the degenerative and metastatic lesions, the ones with the highest SUVmax were selected to determine whether the variations in radiotracer activity were statistically significant and to determine if a cut-off value could help in differentiating between metastatic bone lesions and degenerative bone disease. In the case of metastases, the lesions with the highest radiotracer uptake were identified by using the automatic method generically named “Global Maximum” of the segmentation software, which automatically identifies the voxel with the highest radiotracer uptake. This method can be used several times until the desired number of lesions is achieved. In our case, a minimum of one and a maximum of five metastatic lesions were assessed for every patient. For degenerative lesions, a second method was used that implies identifying a minimum of one and maximum of five of the degenerative lesions with the highest radiotracer uptake by visual assessment. Each area of increased radiotracer activity was correlated with the morphological characteristics of the lesions on the CT image. Characterization of the lesions was made using some of the criteria elaborated by Sapir et al. [27]. To ensure that the selected lesions were, indeed, metastatic or degenerative, we only considered the hypermetabolic foci on SPECT that had a clear correspondent on the CT images. For metastatic lesions, the main criteria were represented by a clear-cut high uptake osteoblastic, lytic, or mixed lesion. In the case of degenerative lesions, the main criteria were represented by accurate overlapping of the region presenting high radiotracer uptake and facet joints, or other degenerative lesions, such as osteophytes. It is important to emphasize that the theoretical gold standard in detection and evaluation of bone metastases is represented by a bone biopsy, but performing a biopsy on every patient would be neither practical nor ethical; therefore, surrogate parameters were used as a reference standard in diagnosis and differentiation between degenerative and metastatic bone lesions. As reported in previous studies, the use of SPECT or SPECT-CT in metastatic bone lesions detection significantly improves the diagnostic sensitivity and sensibility from an AUC index of 0.774 in planar bone scan to an AUC of 0.944 when the bone scan is completed by SPECT [28,29]. Studies conducted by Ahmadzadehfar et al. and San et al. reported a sensitivity and a specificity of 89% and 91%, respectively, of SPECT-CT in bone metastatic lesions detection [23,24].

### 2.4. SUV Calculation

To determine accurate SUV values, multiple parametric data for each patient had to be considered for correction purposes of the SPECT-CT images. The parametric data are represented by patient height, weight, gender, pre-injection activity, the administered activity, post-injection activity, and the exact time at which all the last three parameters were measured. The recording of the exact time of injection is very important because of its correlation with the time of scanning (injection–scan time) at which the SPECT-CT images were acquired. The decay for injected dose and acquisition delay were performed automatically by the dedicated software. The aforementioned parameters, which serve for activity correction and also body weight correction, are completed by the CT-acquired data that represent the baseline for attenuation correction, scatter correction, and resolution recovery performed in the processing phase of the SPECT-CT images. Using advanced segmentation tools that enable accurate definition of the volumes of interest (VOIs) and inherent precision of CT-based organ delineation, quantification SPECT statistics are calculated using the following formula [30]:

SUVlbm = (SPECT image Pixels uptake (Bqml)) × $\frac{\left(\mathrm{LBM}\;\mathrm{in}\;\mathrm{kg}\right)}{\left(\mathrm{actual}\;\mathrm{activity}\right)\cdot 1000}$ units g/mL
for males: LBM in kg = 1.10 × (weight in kg) − 120 × [$\frac{\left(\mathrm{weight}\;\mathrm{in}\;\mathrm{kg}\right)}{\left(\mathrm{height}\;\mathrm{in}\;\mathrm{cm}\right)}{]}^{2}$
for females: LBM in kg = 1.07 × (weight in kg) − 148 × [$\frac{\left(\mathrm{weight}\;\mathrm{in}\;\mathrm{kg}\right)}{\left(\mathrm{height}\;\mathrm{in}\;\mathrm{cm}\right)}{]}^{2}$
actual activity = decay scan × decay1 × (measure activity − (decay2 × post-injection activity))
λ= 0.693 ÷ half lifetime
decay1 = exp (λ × (measured time − administered time))
decay2 = exp (λ × (post-injection time − measured time))
decay scan (injection-scan time) = exp (λ × (administered time-scan time))
LBM in kg = calculated using the dedicated formula for each gender
measure activity = pre-injection activity
measured time = pre-injection time [30]

SUV computation requires quantitative determination of the tracer concentration in target lesions, which necessitates dedicated phantom validation in SPECT-CT absolute quantification using Tc^99m^.

### 2.5. Statistical Analysis of the Data

The data acquired were analyzed using SPSS version 26.0 and MedCalc to perform descriptive analysis, the Shapiro–Wilk test of normality, and the Mann–Whitney nonparametric test, and receiver operating characteristics (ROC) curve analyses were performed. The diagnostic accuracy of the SPECT-CT quantitative analysis was assessed by calculating the area under the ROC curve (AUC). AUC is an overall summary of diagnostic accuracy as follows: an AUC > 0.9 is considered excellent diagnostic accuracy; an AUC between 0.7 and 0.9 is considered good diagnostic accuracy; and an AUC between 0.5 and 0.7 is considered poor diagnostic accuracy. Cut-off values for optimal sensitivity and specificity have been determined by the ROC curves. For all tests, a *p*-value < 0.05 was considered statistically significant.

## 3. Results

### 3.1. Lesions Distribution

**Figure 5:**
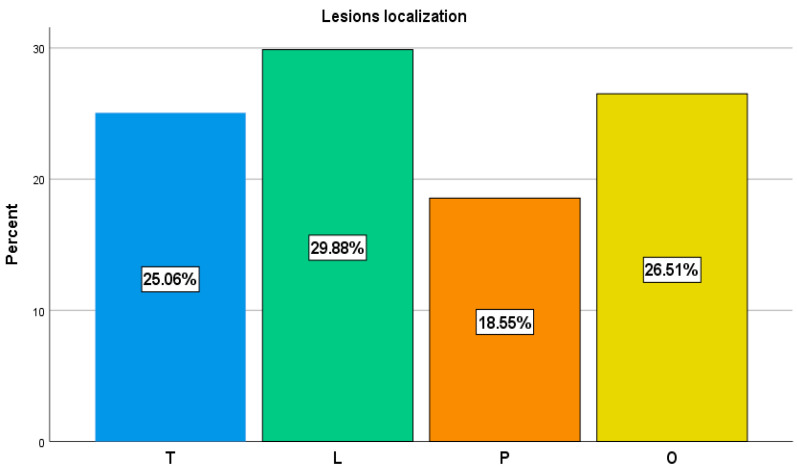
Overall lesions distribution.

A total of 415 lesions were evaluated and categorized as metastatic or degenerative lesions, with the following distribution: 124 (29.88%) lesions in the lumbar vertebrae, 104 (25.06%) in the thoracic vertebrae, 77 (18.51%) in the pelvic bones, and 110 (26.51%) localized in other bone sites (Table 1 and Figure 5).

### 3.2. SPECT-CT SUVmax in Metastatic Lesions and Degenerative Bone Disease

The SPECT-CT data from the 70 patients were assessed using quantitative analysis techniques for each patient, considering the highest values for SUVmax. The number of evaluated lesions varies from one to five lesions for each category. Quantitative data were compared for a total of 415 lesions representing an average of approximately six lesions per patient, with 236 metastatic lesions and 179 degenerative bone diseases. The mean SUVmax for the metastatic lesions (32.56 ± 16.39) ranged from 10.90 to 130.70, while the mean SUVmax of the degenerative bone lesions (10.26 ± 4.67) was between 3.50–27.00 (Table 2).

**Figure 6:**
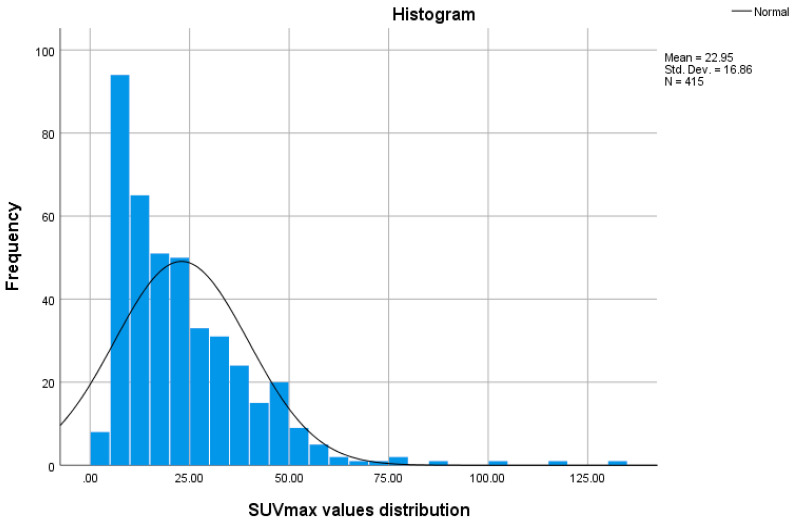
SUVMax values graphic after testing for normal distribution.

**Figure 7:**
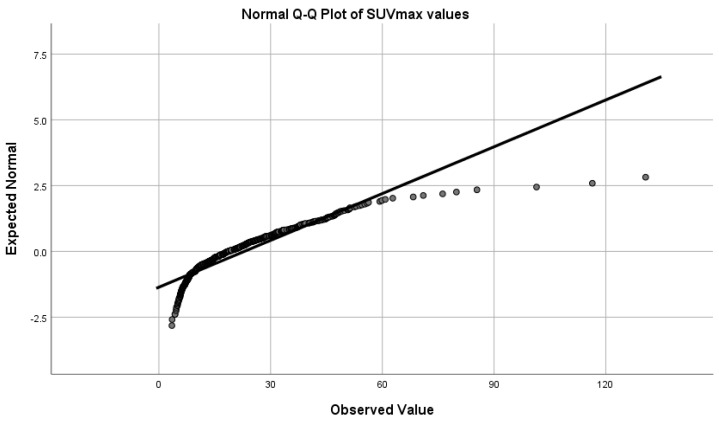
Deviation from normal distribution of the SUVmax values.

Despite registering an overlap of the SUVmax values for the degenerative bone disease lesions and the metastatic lesions, an SUVmax greater than 27.00 always indicated the presence of a metastasis, while a value of less than 10.90 accounted for the presence of a degenerative lesion. To assess if there was any statistically significant difference between the SUVmax of degenerative bone lesions data and the metastatic lesions, we performed a Shapiro–Wilk test of normality (*p* < 0.05 for both degenerative and metastatic lesions), which showed that the data were not normally distributed (Figure 6 and Figure 7); therefore, we used the Mann–Whitney test to determine if any observed difference in the data had a significant difference in the population medians or was simply due to chance, which demonstrated that median value of the SUVmax metastatic lesions is significantly higher than SUVmax value of the degenerative bone lesions (*p* < 0.001).

### 3.3. SUVmax Cutt-off Value in Differentiating between Degenerative and Metastatic Lesions

**Figure 8:**
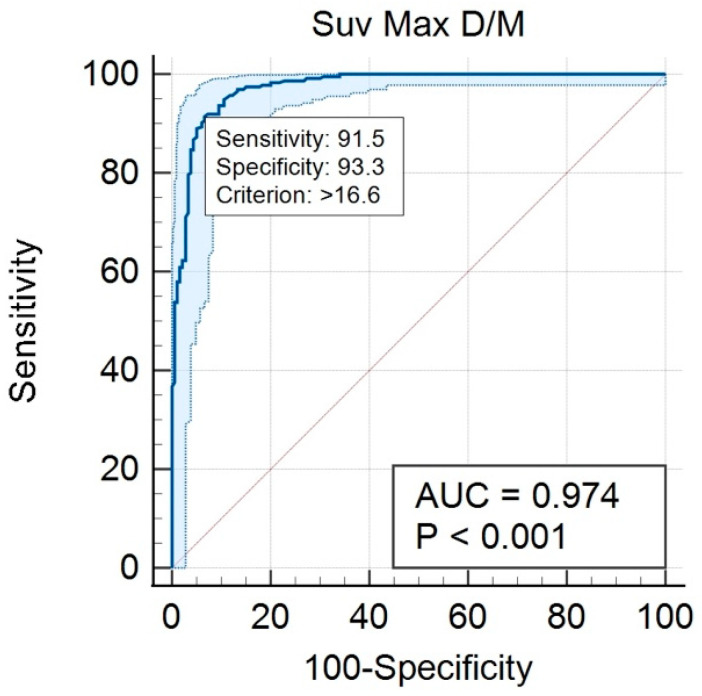
ROC curve analysis.

**Figure 9:**
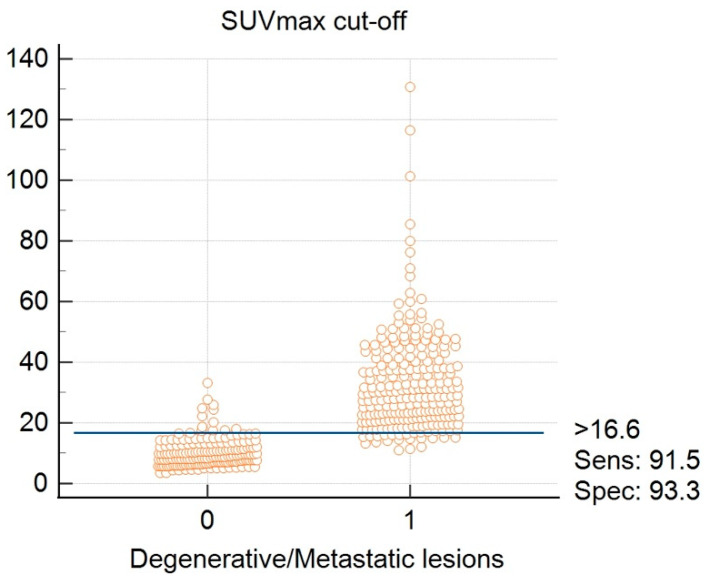
SUVMax cut-off value determination (0—degenerative lesions; 1—metastatic lesions).

To determine the diagnostic accuracy of the SPECT-CT quantitative analysis in differentiating between degenerative bone lesions and metastatic lesions, an ROC curve was drawn. From the ROC curve analysis, a high AUC of 0.974 with a 95% CI of 0.95–0.98 was calculated (Figure 8). From the same statistical test, a cut-off value of SUVmax > 16.6 was identified as being the best point of compromise between sensitivity and specificity, with values of 91.5% and 93.3%, respectively, in discriminating between degenerative and metastatic lesions (Figure 9).

### 3.4. SUVmax Overlapping

**Figure 10:**
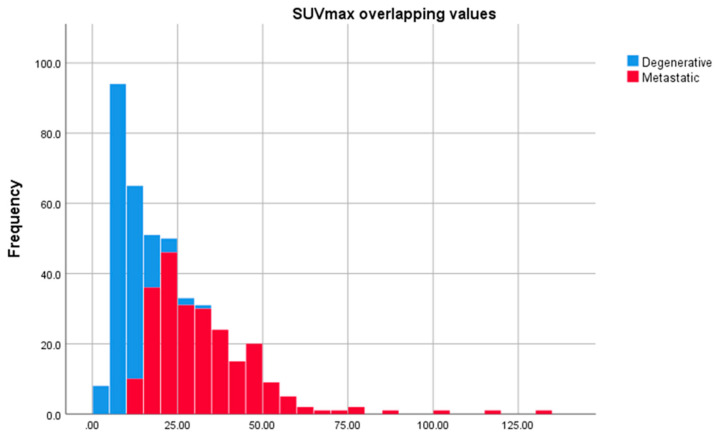
Overlapping SUVmax values in metastatic bone lesions and degenerative bone lesions between 10.90 g/mL and 27.00 g/mL.

Comparison of the SUVmax value between degenerative bone lesions and metastatic lesions resulted in the identification of overlapping values. SUVmax values in the range of 10.90–27.00 represent 67.71% of all SUVmax data, indicating that, in 32.29 % of the cases, a clear difference can be made between the metastatic lesions and the degenerative ones (Figure 10). It is important to mention that, from the total number of 179 degenerative lesions, only two lesions (1.1%) had an SUVmax value of 27.00, five lesions (2.8%) ranged between 20.00–26.99, and 172 lesions (96.1%) were below 20.00. The 236 metastatic lesions evaluated had the following distribution using the same criteria: 132 lesions (55.9%) had an SUVmax value greater than 27.00, 58 lesions (24.6%) ranged between 20.00–26.99, and 46 lesions (11.1%) had an SUVmax value below 20.00. These data show that the high percentage of overlapping values results from the small number of degenerative lesions (seven lesions) that ranged between 20.00–27.00.

## 4. Discussion

Given the fact that bone tissue represents the most common site of secondary lesions development in stage IV breast cancer patients, the detection of the metastases and their clinical management is of great importance [31]. The main role of molecular imaging techniques, such as bone scintigraphy and SPECT-CT, is to determine the accurate diagnosis of bone metastasis [32]. The highest sensitivity for evaluating the presence of metastatic bone lesions in breast cancer patients was shown by F^18^-NaF-PET-CT, followed by ^99m^Tc-MDP, and then FDG-PET-CT [33].

To the best of our knowledge, this is the first study to report the quantification of ^99m^Tc-HDP uptake in bone metastases of breast cancer patients alone, using the SUVmax values for degenerative and metastatic lesions from the same patient. Relatively similar studies, conducted by Beck et al. [22] and Arvola et al. [18], included both prostate and breast cancer patients. Although Arvola et al. performed quantitative analysis on metastatic breast cancer patients, their goal was to determine the degree of correlation between the SUVs resulting from Tc^99m^-HDP SPECT-CT and ^18^F-NaF PET-CT studies. The results indicated a high degree of correlation between the two methods [18], showing that SPECT-CT SUVs are feasible for uptake measurement in bone metastasis.

Another SPECT-CT quantitative analysis calculation performed by Beck et al. included only 19 patients, compared to 70 patients in our study, and a significantly smaller number of metastatic lesions were evaluated: 52 compared to 236, registering a mean SUV peak value of 20.4 ± 20.8, compared to the mean SUVmax value 32.56 ± 16.39 in our study. The main goal of Beck et al. was to determine the degree of inter-method agreement between the visual and quantitative assessment of disease progression, while reporting the radiotracer uptake in absolute units [22]. A similar SPECT-CT study was performed by Rohani et al. by evaluating bone metastasis in 34 prostate cancer patients with overall data close to that derived from the present one. The total number of lesions evaluated by Rohani et al. was 215, compared to 415 in our study, with 89 vs. 179 degenerative lesions and 122 vs. 236 metastatic lesions, thus improving the statistical significance of the resulting data from the present study compared to theirs. The mean value in degenerative bone lesions registered by Rohani et al. was 12.59 ± 9.01, compared to 10.26 ± 4.67, and the mean value for metastatic bone lesions was 36.64 ± 24.83, compared to 32.56 ± 16.39, representing fairly close values. The most notable differences in the data gathered from the two studies are represented by the cut-off value of ≥ 20 g/mL from Rohani et al. and 16.6 g/mL determined by our data, in differentiating metastatic bone lesions from the degenerative ones, and the overlapping values of the degenerative and metastatic lesions of 6.37–49.33 compared to 10.90–27.00 [32]. A retrospective study conducted by Kuji et al. on 170 patients with prostate cancer was evaluated, which registered higher SUVmax values compared to the ones obtained from our study in terms of the mean value for degenerative bone lesions (16.73 ± 6.74 compared to 10.26 ± 4.67) and metastatic bone lesions (40.90 ± 33.46 compared to 32.56 ± 16.39) [21].

It is crucial to mention that both Rohani et al. and Kuji et al. used SUV normalized by body weight (SUVbw) to assess the bone lesions, while in our case SUV normalized by lean body mass (SUVlbm) was the method of choice. Based on reports in the literature, the SUVbw is affected by the amount of body fat; therefore, the SUV values are overestimated in obese patients, while SUVlbm is not subjected to variations due to body weight or by the amount of lean body mass [34]. Further, for accurate quantitative measurement purposes, the parameters used for imaging reconstruction play an important role as the number of subsets and iterations can change the SUV values, with the main principle being that the higher the number of iterations and subsets, the more accurate the measurements are [35]. In our case, we used 10 iterations with eight subsets, compared to eight iterations and four subsets used by Beck et al. The data from Kuji et al. and Rohani et al. on the number of iterations and subsets used were not available for comparison.

Considering the data found in the literature, with the emphasis on those cited in this article, although metastatic lesions from breast cancer are naturally more lytic than the metastatic lesions from prostate cancer, their avidity for ^99m^Tc-HDP is comparable, which is relatively peculiar, given the known fact that osteolytic lesions tend to have less ^99m^Tc-HDP uptake than the osteoblastic ones. This study, together with the ones conducted by Kuji et al. [21] and Rohani et al. [32], showed a statistically significant difference in SUVmax value between degenerative bone lesions and metastatic bone lesions where, as expected, higher values were recorded for metastatic lesions. The difference can be explained by the fact that the metastatic breast cancer cells can alter the normal biological processes involved in bone remodeling molecular mechanisms [36]. To assess the diagnostic accuracy in differentiating degenerative lesions from metastatic lesions, an ROC curve analysis was performed. In our study, the AUC had a high value of 0.974 (95% CI 0.95–0.98), more than the values registered by Kuji et al. and Rohani et al. (0.974 vs. 0.926 and 0.874, respectively). A significantly higher sensitivity resulted from our study compared to Rohani et al. (91.5 % vs. 73.8%), also with comparable specificity (93.3% vs. 85.4%). Unfortunately, the sensitivity and specificity values of the Kuji et al. study were not available for comparison. Our results suggest that the SUVmax value resulting from the quantitative analysis of the SPECT-CT data in breast cancer patients with metastatic bone disease can help in discriminating active bone metastases from degenerative bone lesions whenever a clear diagnosis cannot be determined based on the acquired images, thus improving the diagnostic accuracy and the patient’s clinical management. It is important to mention that, although the quantitative analysis can help the bone scan interpretation, offering a more standardized method and reducing the interobserver differences, the main application of SPECT-CT data quantification could be represented by patient follow-up and interpatient comparison.

### Limitations

The limitations of this study are represented by: (1) in case of a degenerative lesion localized next to a metastatic lesions, in the same region of interest we could not discriminate the uptake in the degenerative one and, therefore, it is not possible to say without doubt that the selected lesions were, indeed, the ones with the highest uptake in every patient; (2) the spatial resolution of SPECT and the difficulty in assessing small lesions; (3) we only considered lesions that had a clear correspondent on the CT images to ensure that the selected lesion was certainly metastatic or degenerative; but, in some cases, hypermetabolic lesions with no correspondent on the CT images were not evaluated; (4) the data were determined based on a single data set and were not applied to other validation sets; and (5) SUV computation errors due to scanner calibration and partial volume effect.

## 5. Conclusions

In conclusion, quantitative analysis of the SPECT-CT data can improve accuracy in discriminating degenerative bone lesions from metastatic lesions. The SUV max cut-off value of 16.6 g/mL obtained through ROC curve analysis can help to discriminate metastatic from degenerative lesions in breast cancer patients, with a sensitivity of 91.5% and a specificity of 93.3%. Treatment effectiveness evaluation, patient follow-up, and interpatient comparison represent further benefits in performing SPECT-CT SUV calculation. Further extensive studies need to be conducted in order to be able to use these results in clinical practice.

## Figures and Tables

**Table 1:** Localization of metastatic and degenerative lesions.

Region	No. of Lesions	No. of Metastatic Lesions	No. of Degenerative Lesions
Lumbar vertebrae (L)	124	50	74
Thoracic vertebrae (T)	104	50	54
Pelvic bones (P)	77	61	16
Other bone sites (O)	110	75	35

**Table 2:** Mean values of the SUVMax in degenerative and metastatic lesions.

SUVMax Value	No.	Min.	Max.	Mean	Std.dev.
Metastatic lesions	236	10.90	130.70	32.56	16.39
Degenerative lesions	179	3.50	27.00	10.26	4.67

## Data Availability

All the data generated or analyzed during this study are included in the manuscript.
